# Impact of In Vitro Gastrointestinal Digestion on the Bioaccessibility of Phytochemical Compounds from Eight Fruit Juices

**DOI:** 10.3390/molecules26041187

**Published:** 2021-02-23

**Authors:** Dasha Mihaylova, Ivelina Desseva, Magdalena Stoyanova, Nadezhda Petkova, Margarita Terzyiska, Anna Lante

**Affiliations:** 1 Department of Biotechnology, Technological Faculty, University of Food Technologies, 4002 Plovdiv, Bulgaria; dashamihaylova@yahoo.com; 2 Department of Analytical Chemistry and Physical Chemistry, Technological Faculty, University of Food Technologies, 4002 Plovdiv, Bulgaria; magdalena.stoianova@abv.bg; 3 Department of Organic Chemistry and Inorganic Chemistry, Technological Faculty, University of Food Technologies, 4002 Plovdiv, Bulgaria; petkovanadejda@abv.bg; 4 Department of Informatics and Statistics, University of Food Technologies, 4002 Plovdiv, Bulgaria; mterziyska@uft-plovdiv.bg; 5 Department of Agronomy, Food, Natural Resources, Animals, and Environment-DAFNAE, Agripolis, University of Padova, 35020 Legnaro, Italy

**Keywords:** bio-accessibility, in vitro digestion, antioxidants, phytochemicals, fruit juice

## Abstract

Fruits contain a number of useful substances including antioxidants. Their bio-accessibility after passing through the digestive tract is of primary importance when considering their benefits. In this respect, we investigated the effect of in vitro digestion on the phytochemicals of eight fruit juices. Freshly prepared juices from pomegranate, orange and grapefruit were used as well as commercially available juices from cherry, black grapes and aloe vera, blackberry and chokeberry, and two types of chokeberry and raspberries. Spectrophotometric and HPLC methods were used in order to analyse the sugar content, the total phenolic (TPC) and flavonoid contents (TFC), anthocyanins, phenolic acids and antioxidant activity. Principle component analysis was used to explain the differentiation among the types of fruit juice. Sugar recovery variation was between 4–41%. The bio-accessibility of TPC ranged from 13.52–26.49% and of flavonoids between 24.25–67.00%. The pomegranate juice and the juice of black grapes and aloe vera kept 58.12 and 50.36% of their initial anthocyanins content, while for the other samples less than 1.10% was established. As a result, a maximum of 30% remaining antioxidant activity was measured for some of the samples, but for most this was less than 10%. In conclusion, fruit juices are a rich source of biologically active substances, but a more detailed analysis of food transformation during digestion is needed.

## 1. Introduction

Biological antioxidants have been defined as “compounds that protect biological systems against the potentially harmful effects of processes or reactions that cause extensive oxidations” [1]. Many food components are thought to possess antioxidant activity and are therefore considered to be beneficial for human health. Although a huge number of papers is focused on antioxidants research annually, there is still more to explore.

According to The World Health Organization [2] a daily consumption of at least 400 g of fruits and vegetables (equivalent to five daily servings) decreases the risk of several noncommunicable diseases: cardiovascular, chronic, and other diseases. The reason for this is their diverse composition. As plant derivatives, fruits and vegetables are rich in different secondary metabolites such as phenolic compounds, saponins, alkaloids, terpenoids, etc. [3]. The presence of these compounds is a prerequisite for health-promoting effects. Antioxidant, antibacterial, antifungal, antiviral, cholesterol-lowering, antithrombotic, or anti-inflammatory effects are among the most cited [3,4]. Accordingly, fruits and vegetables are very important in the human diet.

Food digestion is a complex process with many factors involved including individual particularities, resulting in expensive in vivo experiments and complicated data. In the past 10 years, much effort was put into establishing a reliable approach for omnibus human digestion simulation. Although this is a challenging task, several methods were proposed, with that of Brodkorb et al. [5] being the most comprehensive. Certainly, in vitro digestion models do not cover all the complexity of the process, but they are considered useful in predicting in vivo digestion impact [6]. Gastric digestion and intestinal digestion are expected to have different effects on phytochemicals, and thus change their antioxidant activity [7,8]. Previous studies have revealed that in some cases the content of phenolic substances and their antioxidant activity increased [9,10,11], while in others a decrease is reported [12,13].

It has to be noted that various processing methods applied to food materials may have significant effects on their antioxidant potential and phytochemical bio-accessibility. For instance, the bio-accessibility and bioavailability of phenolic compounds could be affected by interaction with other present macromolecules such as proteins, carbohydrates and lipids [14]. These interactions could protect the phenolic compounds from oxidation during their passage through the gastrointestinal tract. On the other hand, phenol/protein interactions can lead to a loss of nutritional value due to protein precipitation and enzyme inactivation [15]. In this respect, more detailed phytochemical characteristics and more complete understanding of the digestion processes are needed [16].

So far, there are few reports on the effects of simulated digestion on the phenolic compounds and antioxidant activities of fruit juices [17]. Therefore, the aim of the present study is to evaluate the effect of in vitro digestion on the content and composition of sugars and phenolic substances of eight either freshly cold-pressed or commercially available fruit juices. The remaining antioxidant activity was also studied, thus revealing the real benefits for the human body of juice consumption. In this regard, spectrophotometric and HPLC (High-performance liquid chromatography) methods were used in order to evaluate the phytochemical profile and bio-accessibility.

## 2. Results and Discussion

Fruits and fruit juices are widely studied with respect to their biologically active substances and antioxidant capacity [18,19,20], but not many studies have focused on the transformations that occur during digestion [10,13,21]. The potential bioavailability of biologically active phytocompounds is important information linked directly to their health claims [22,23,24,25]. In this regard, physicochemical characteristics, phytochemical profile and the bio-accessibility of sugars and phytochemicals in eight fruit juices were assessed.

### 2.1. Physicochemical Characteristics, Individual and Total Sugar Content of Fruit Juices Prior to In Vitro Digestion

In this study eight fruit juices were subjected to in vitro gastrointestinal digestion. The individual and total phytochemical changes were studied, as well as the remaining antioxidant activity. Table 1 presents the results of the preliminary analyses of the tested juices. Naturally, fruit juices have a low pH [26]. This is mostly related to the presence of organic acids. The pH of the studied samples was between values 3 and 4, with grapefruit juice displaying the lowest (3.08) and pomegranate juice the highest value (3.80). The dry matter content varied from 5.1–16.67%, and the highest value was established in sample C (grapefruit juice) and the lowest in sample H (chokeberry and raspberry).

Fruit juices are rich sources of sugars. In this regard, the individual content of sucrose, glucose, fructose and sorbitol was assessed by the HPLC-RID (refractive index detector) method, while the total sugar content was evaluated by the phenol-sulphuric method (Table 1). The results from HPLC-RID analysis indicated that the monosaccharides glucose and fructose dominated in all initial juices. Similar findings were reported earlier for carbonated beverages and fermented milks [27]. Тhe total sugar content in the studied juices varied between 2.69 and 10.51 g/100 g juice. The lowest value was detected in sample H, which, however, is enriched with the sweetener sorbitol and steviol glycosides. Sorbitol was only detected in the samples that contained aronia juice (Sample D, E and H), because it is inherent in this fruit [28]. In sample H 0.90 g/100 g juice sorbitol were detected, while in samples D and E its content was 0.49 and 0.58 g/100 g juice, respectively.

### 2.2. Phytochemical Profile of Fruit Juices Prior to In Vitro Digestion

The total phenolics, total flavonoids and total monomeric anthocyanins content of the studied fruit juices were evaluated and the results are presented in Table 2. The TPC varied between 0.81 ± 0.01 mgGAE/mL for sample G and 15.56 ± 0.06 mgGAE/mL for sample A. Tenfold lower content of extracts of citrus fruits was reported by Fejzić and Ćavar [29]. In the literature it is well demonstrated that chokeberry is one of the richest fruits in polyphenols [30]. In this regard, our results are in agreement with that of Denev et al. [31]. The lowest initial TFC was measured for sample G (79.43 ± 3.39 μgQE/mL), while the highest was for sample C (1667.71 ± 23.66 μgQE/mL). Similar TPC results are reported by Wern et al. [32], who studied fresh fruit juices, commercial fruit juices and fruit drinks. The total monomeric anthocyanins varied among the samples between 0 and 42.44 ± 0.52 mg/L. The highest content was detected in sample H. No anthocyanins were detected in samples B and C as they naturally do not possess such.

The content of phenolic acids evaluated by HPLC method was also studied and the results are presented in Table 3. Rosmarinic, cichoric and cinnamic acids were not detected in any of the samples and are not included in the table. Sample C displayed the highest content prior digestion for p-coumaric (12.23 ± 0.11 µg/100 g juice) and sinapic (8.45 ± 0.11 µg/100 g juice) acids and sample D for caffeic acid (3.25 ± 0.01 µg/100 g juice).

### 2.3. Bioaccessibility of Phytochemicals after In Vitro Digestion

**Figure 1:**
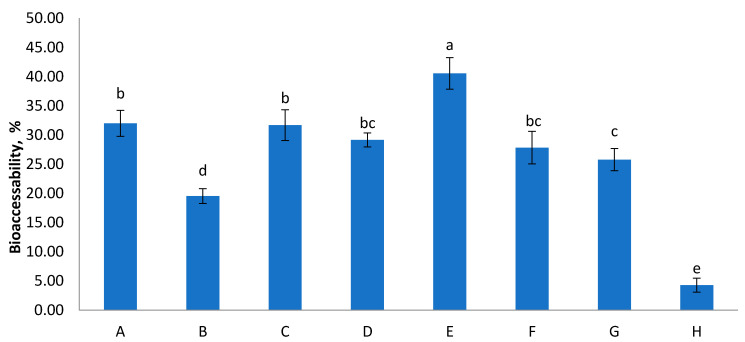
Bio-accessibility of total sugars from the digested juices (%). Values are means ± SEM, *n* = 3 per treatment group. Means in a chart column without a common superscript letter differ (*p* < 0.05) as analysed by one-way ANOVA and the TUKEY test.

The physiological conditions, such as pH, the presence of digestion enzymes and bile salts, significantly affect the bio-accessibility of food components [33] and their evaluation will contribute to understanding their biological significance within functional products [34,35]. Bio-accessibility of total sugars in foods significantly contributes to sugar intake [27]. Figure 1 presents the results of sugar availability after in vitro digestion of the studied samples. The recovery varied between 4–41% between samples. The lowest bio-accessibility was reported for sample H. Our observations were comparable with findings for other food products reported by Choi et al. [27]. According to their reports bio-accessibility of total sugars in carbonated beverages ranged from 54.6–69.4%, and in fermented milks from 11.8–85.0%. In the observation of patterns of sugar released from food matrices, both fructose and glucose are major compounds that are bioaccessible, implying that these are potentially bioavailable or susceptible to absorption through the gut barrier. The possible explanation for this common bio-accessibility of sugars from fruit juices are the complex food matrix and the presence of polyphenols and tannins, especially in red berries [36,37]. It has been reported previously that polyphenols may have an inhibitory effect on digestive enzymes such as α-amylase and α-glucosidase. Moreover, tannic acid and catechins decreased in vitro digestibility of various types of starch sources [38].

Simulated digestion had a significant negative impact on the total polyphenol content of the tested fruit juices (Table 4). The resulting bio-accessibility ranged between 13.52–26.49%. The highest percentage was calculated for sample F, followed by sample D. It has been found by Bermudez-Soto et al. [39] that polyphenols are highly sensitive to mildly alkaline conditions such as in the intestine, where a proportion of the compounds is transformed into different structural forms with different chemical properties. In addition, during digestion phenolic compounds are susceptible to interaction with other released food components, such as iron, other minerals, dietary fibre or proteins that can lead to loss of phytochemicals [40].

Flavonoids were less influenced by the digestion process resulting in bio-accessibility ranging between 24.25% (Sample D) and 67.00% (Sample F). Ovando-Martnez et al. [41] reported that the TFC of red chiltepin decreased after digestion in simulated gastrointestinal fluid, which was consistent with the present study.

The simulated digestion procedure included acidic pH (3.0) in the gastric phase, and subsequent increase to mildly alkaline pH (7.5) in the intestinal phase resulted in a significant decrease in the total monomeric anthocyanins present in the juices (Table 3). This transition leads to a reduction in the number of bio-accessible anthocyanins, as these pigments are highly unstable at intestinal pH. However, relatively moderate bio-accessibility was detected for samples A and G, where the initial content had the highest values. The bio-accessibility in these samples was 58.12% and 50.36%, respectively. For samples D, E, F, and H this was less than 1.10%.

The bio-accessibility of the phenolic acids in the fruit juices was also affected by the digestion process and the results are presented in Table 5. Both loss and gain of phenolic acids were noted. For all the three freshly prepared juices, samples A, B, and C, chlorogenic acid was partly lost, resulting in bio-accessibility of 49.52%, 9.20%, and 20.06%, respectively. Meanwhile, in 4 of 5 commercial juices (samples D, E, and H) this acid was not detected in the initial juices, but was released after the digestion procedure. Our results are in agreement with Bermúdez- Soto et al. [39] who reported increase of chlorogenic acid content after digestion of chokeberry, probably due to isomerisation reactions of neochlorogenic acid. While gallic acid was not quantifiable in sample D prior to digestion, 26.96 ± 0.23 µg/100 g were detected afterwards. The same tendency was observed for protocatechuic acid in sample F, resulting in 20.70 ± 1.01 µg/100 g after treatment. p-Coumaric acid was also liberated throughout digestion in samples A and E (2.60 ± 0.08 and 5.06 ± 0.04 µg/100 g, resp.), and sinapic acid in E and F (2.00 ± 0.03, and 1.11 ± 0.01 µg/100 g, respectively). In this regard, other researchers considered the phenolic compounds as highly bio-accessible and potentially bioavailable with high stability. Seraglio et al. [42] reported a significantly increased bio-accessible fraction of phenolic compounds after simulated digestion when analyzing honey (1.73 times more). This may be due to the highly chemo-divergent nature of phenolic compounds, that include simple molecules on the one hand and big polymers on the other [43]. The latter are hydrolyzed during digestion as a sequence of the acidic environment of the stomach and of the slightly alkaline conditions of the intestine, as well as the action of the digestive enzymes [11]. These conditions lead to several changes in the phenol structure such as hydroxylation, methylation, iso-prenylation, dimerization and glycosylation, as well as formation of phenolic derivates by partial degradation of the combined forms due to losing the moieties between phenols and sugars [44].

### 2.4. Antioxidant Capacity before and after In Vitro Gastrointestinal Digestion of Fruit Juices

**Figure 2:**
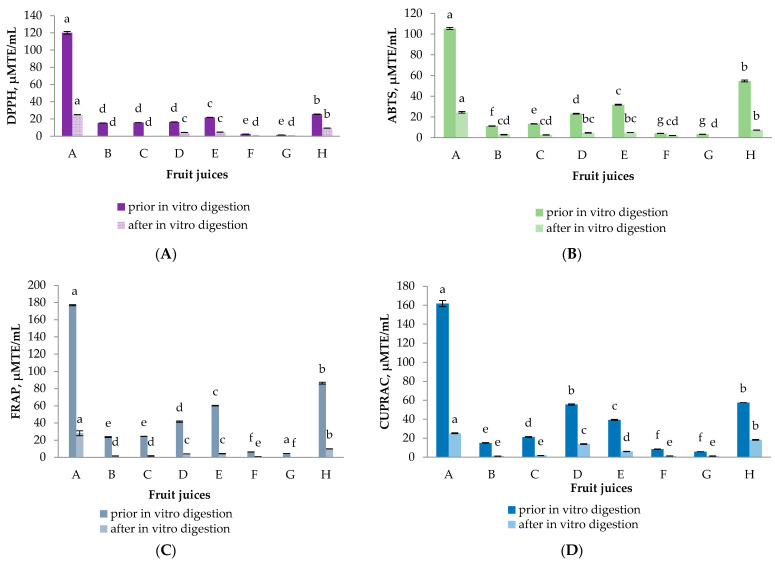
Antioxidant capacity of selected fruit juices before and after in vitro gastrointestinal digestion by (**A**) 2,2-diphenyl-1-picrylhydrazyl (DPPH) radical scavenging activity, (**B**) 2,2′-azino-bis(3-ethylbenzothiazoline-6-sulfonic acid (ABTS) radical scavenging activity, (**C**) Ferric-Reducing Antioxidant Power (FRAP) and (**D**) Cupric Ion Reducing Antioxidant Capacity (CUPRAC) assays. The data is presented as the mean (*n* = 3) ± S.D. Different letters within chart columns indicate significant differences between treatments according to Tukey’s test at *p* < 0.05.

The presence of phytochemicals in the tested fruit juices predetermines their antioxidant activity (AOA). In this regard four generally recognized methods were used (Figure 2). The information they provide is complementary, as these methods differ in their mode of action. Ferric-Reducing Antioxidant Power (FRAP) and Cupric Ion Reducing Antioxidant Capacity (CUPRAC) assays evaluate the reducing potential of the sample, whereas 2,2-diphenyl-1-picrylhydrazyl (DPPH) and 2,2′-azino-bis(3-ethylbenzothiazoline-6-sulfonic acid (ABTS) assays evaluate its anti-radical scavenging activity [45]. While it may be useful to know the antioxidant capacity of fruit juices before digestion for comparative purposes, this is not a true reflection of their potential health effects. A more realistic view is gained from the antioxidant capacity after the fruit juices have undergone a simulated digestion procedure, when the antioxidants potentially available for absorption can be measured. In our study, the highest initial activity was established for samples A and H. For these two samples as well as for sample D and E the reducing potential was higher compared to the anti-radical scavenging activity. The results were in accordance with the highest TPC of these juices indicating the main contribution of the polyphenolic compounds to the antioxidant potential of the samples. Ryan and Prescot [46] also determined that pomegranate juice possessed the highest antioxidant activity pre- and post-digestion compared to other types of juice that were analyzed (orange, apple, pineapple, grapefruit, red grape, cranberry). It seems that the TFC did not contribute to the overall AOA of the samples. Although sample C displayed the highest TFC prior to digestion, the initial antioxidant potential of this sample was negligible and almost nil after digestion. The loss of phytochemicals reflects the remaining antioxidant capacity after digestion [11]. This trend was observed for all the samples by all the methods used. A maximum of 30% remaining AOA was measured for some of the samples. However, for the majority of digested juices less than 10% was detected. Our results are consistent with previous research studies on fruit juices subjected to digestion, where significant decreases in antioxidant capacity were demonstrated [10,13,39,47].

### 2.5. Principal Component Analysis

Principal component analysis (PCA) allows summarizing of information in large data tables by means of a smaller set of factors in such a way as to be more easily visualized and analyzed. Another kind of graphic closely associated with PCA is biplot. This shows the variances and correlations of the variables, as well as the distances among the units. Biplot consists of lines and dots. Lines are used to reflect the variables of the dataset, and dots are used to show the observations.

**Figure 3:**
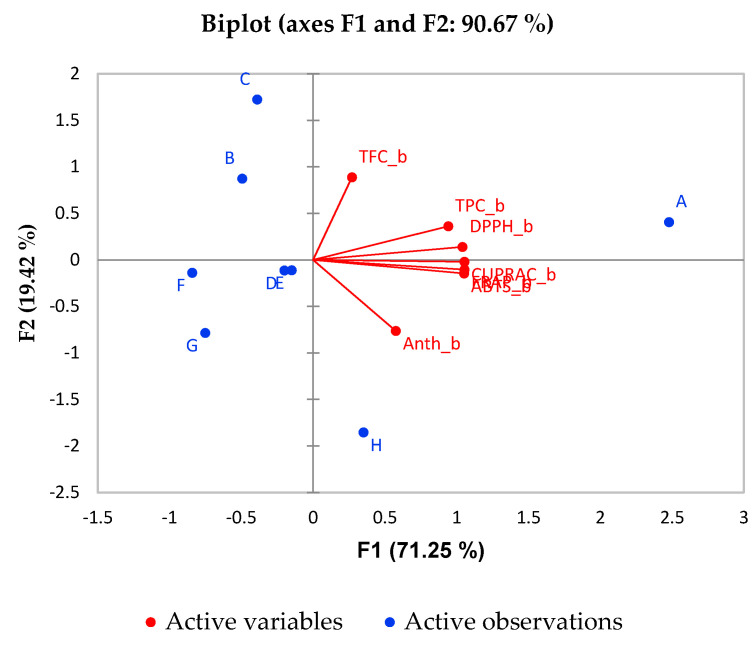
Biplot of for PCA analysis of fruit juices.

**Figure 4:**
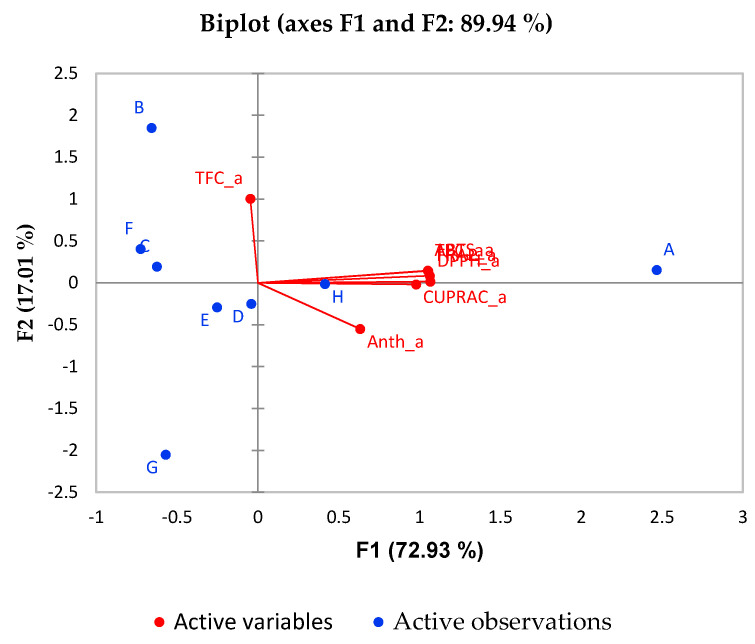
Biplot for PCA analysis of in vitro digested juices.

In the current study, PCA is used to explain the differentiation among the different types of fruit juice by a small number of linear combinations of the different variables responsible for most of the variability in the data. The PCA analysis was performed under the following conditions: the Kaiser-Meyer-Olkin Measure of Sampling Adequacy statistic is 0.741, which exceeds the recommended value of 0.60. Bartlett’s test for sphericity was statistically significant (*p* < 0.001). Here, the studied dataset are eight different types of juice, and the variables are their chemical characteristics: TPC, TFC, Anthocyanins and AOA. The biplot graphics based on PCA for the most important factors (F1 and F2) for prior to digestion and post-digestion stages are given in Figure 3 and Figure 4, respectively. It can be seen from Figure 3 that TPC contributes significantly towards the AOA of most of the juices. The percentage of variability represented by the first two factors is high (90.67%) in the case of prior digestion. The first factor (F1) explains 71.25% of the total variance of the significant parameters TFC and AOA, whereas F2 explains 19.42% of the total variance with significant parameters TPC and Anthocyanins in the phase prior in vitro digestion. From the PCA analysis, the pomegranate juice (sample A) was found to be more associated with antioxidant activity and phenolic compound content prior to the digestion phase.

It is evident from Figure 4 that the trend is almost the same for the in vitro post-digestion phase. The percentage of variability represented by the first two factors is high (89.94%) in this case. The first factor (F1) explains 72.93% of the total variance of significant parameters TPC and AOA of the different juices while F2 explains 19.01% of the total variance of significant parameter TFC. Again, sample A (pomegranate juice) is associated with the best antioxidant activity and phenolic compounds content. Interestingly, in sample H better correlation with the AOA and phenolic compounds is observed after digestion compared to prior in vitro digestion.

## 3. Materials and Methods

### 3.1. Chemicals and Reagents

All reagents used in this study were of analytical grade and purchased from Merck Chemicals (Darmstadt, Germany) and Sigma-Aldrich (St. Louis, MO, USA).

### 3.2. Fruit Juices Samples

The fruit juices that were used in this study were either purchased from a local fresh fruit juice shop (three samples) where they were freshly cold-pressed on a slow-turn juicer or purchased from a local shop as randomly chosen commercial products (five samples). The cold pressed juices were obtained from pomegranate (*Punica granatum* L., sample A), orange (*Citrus* × *sinensis* (L.) Osbeck, sample B) and grapefruit (*Citrus* × *paradisi* Macfad, sample C). The commercial products on the other hand were mixtures as follows: juice of raspberries and chokeberry (3:1, min 40% fruit content, sample D), juice of blackberry and chokeberry (3:1, min 40% fruit content, sample E), juice of cherry (min 40% fruit content, sample F), juice of black grapes and aloe vera juice and chunks (10 and 12%, respectively, sample G) and juice of chokeberry and raspberries (36 and 4%, respectively, with natural sweetener steviol glycosides, sample H). All juices were then immediately subjected to analysis. Three independent samples were made and tested from the same material and the results are presented as mean. Simulated gastric fluid (SGF) and simulated intestinal fluid (SIF) were prepared as described by Brodkorb et al. [5].

### 3.3. In Vitro Gastro-Intestinal (GI) Digestion

The assay was performed according to the procedures described by Brodkorb et al. [5] with minor modifications. Only the gastric and intestinal phase were included.

#### 3.3.1. Gastric Phase

Fruit juice (5 mL) was mixed with 3.62 mL of a porcine pepsin stock solution (pepsin from porcine gastric mucosa, P7000, Sigma-Aldrich, St. Louis, MO, USA; 5520 U/mL made up in SGF electrolyte stock solution), 2.5 μL of 0.3 M CaCl_2_ and 132 µL of phospholipids (0.17 mM in the final digestion mixture). The pH of the mixture was corrected with 1 M HCl to the value of 3.0 and the volume of the mixture was made up to 10 mL with distilled water. The mixture was then incubated at 37 °C with constant shaking in a shaking water bath for 2 h. The pH was regularly checked and re-adjusted with 1 M HCl when needed.

#### 3.3.2. Intestinal Phase

Gastric chyme (10 mL) was mixed with 8 mL of a pancreatin solution (pancreatin from porcine pancreas, P1750, Sigma-Aldrich, St. Louis, MO, USA; 1.72 U/mL made up in SIF electrolyte stock solution based on trypsin activity), 1.9 mL of fresh bile extract (160 mM fresh bile salts in final mixture, Sigma-Aldrich, St. Louis, MO, USA), 20 μL of 0.3 M CaCl_2_, 1 M NaOH to reach pH 7.5, and water to 20 mL total volume. The mixture was then incubated at 37 °C in a shaking water bath for 2 h. The pH was regularly checked and re-adjusted with 1 M NaOH during the process if needed.

For the blank sample, water was used instead of juice. The values obtained for blanks were subtracted from the sample values for each analysis. The digestion sample was then centrifuged and stored at −20 °C till further analysis, but no longer than for 7 days.

### 3.4. Moisture Content

Total moisture content of the samples was determined in moisture analyzer balance (Radwag PMC 50/NH, Radom, Poland). The sample was placed in a dish and dried to constant mass at 105 °C.

### 3.5. Total Polyphenol Content Analysis (TPC)

The total polyphenol content was analyzed using the Folin-Ciocalteu method of Kujala et al. [48] with some modifications. Each sample (1 mL) was mixed with 5 mL of Folin-Ciocalteu’s phenol reagent and 4 mL of 7.5% Na_2_CO_3_. The mixture was vortexed for 10 s and incubated for 5 min at 50 °C. The absorbance was then measured at 765 nm against a blank consisting of solvent instead of sample, using SPECTROstar Nano Microplate Reader (BMG LABTECH, Ortenberg, Germany). The TPC in the extracts was expressed as mg gallic acid equivalent (GAE) per mL juice. The linear range for gallic acid standard was 5–100 mg/L (*R*^2^ = 0.9965).

### 3.6. Total Flavonoid Content (TFC)

The total flavonoid content was evaluated according to the method described by Kivrak et al. [49]. An aliquot of 0.5 mL of native or digested juice samples was added to 0.1 mL of 10% Al(NO_3_)_3_, 0.1 mL of 1 M CH_3_COOK and 3.8 mL of ethanol. After incubation at 22 °C for 40 min, the absorbance was measured at 415 nm against a blank consisting of solvent instead of sample using SPECTROstar Nano Microplate Reader (BMG LABTECH, Ortenberg, Germany). Quercetin was used as a standard in the linear range of 5–80 μg/mL (*R*^2^ = 0.9972) and the results were expressed as mg quercetin equivalents (QE)/mL juice.

### 3.7. Total Monomeric Anthocyanin Content

The total monomeric anthocyanin content was determined using the pH- differential method [50]. Properly diluted samples were mixed separately with KCl (0.025 M, pH 1.0) and CH_3_COONa (0.4 M, pH 4.5) in 1:4 ratio. The absorbance (A) was measured using SPECTROstar Nano Microplate Reader (BMG LABTECH, Ortenberg, Germany) at 520 and 700 nm after 15 min incubation at 22 °C against a blank consisting of distilled water instead of sample, and the results were calculated as follows:

A = (A_520_ − A_700_)pH 1.0 − (A_520_ − A_700_)pH 4.5 (1)

The monomeric anthocyanin (MA) pigment concentration in the samples was calculated as:

Monomeric anthocyanin pigment (mg/L) = (A × MW × DF × 1000)/(ε × 1)(2)

where M represents the molar mass of cyanidin-3-glycoside (449.2 g/M), DF is the dilution factor, ε is the molar extinction coefficient (26,900 L/M × cm), and 1 is the cuvette optical path length (10 mm). The final anthocyanin concentration of native or digested juice is expressed as µg cyanidin-3-glucoside (C3GE)/L juice.

### 3.8. Determination of Antioxidant Activity

#### 3.8.1. DPPH• Scavenging Activity

The ability of the sample to donate an electron and scavenge DPPH radicals was determined by the slightly modified method of Brand-Williams et al. [51]. Freshly prepared 4 × 10^−4^ M solution of DPPH radicals was mixed with sample in a ratio of 2:0.5 (*v/v*). The absorption was measured at 517 nm after 30 min incubation at 22 °C against a blank (distilled water). The absorbance of a control sample, a solution prepared in the same manner but with water instead of sample, was also measured using SPECTROstar Nano Microplate Reader (BMG LABTECH, Ortenberg, Germany). The DPPH radical scavenging activity of native or digested juice was presented as Trolox equivalents (TE) in the linear range of the standard 50–500 μM/L (*R*^2^ = 0.9985) and expressed as μM TE per mL of juice (μM TE/mL).

#### 3.8.2. ABTS^•+^ Scavenging Activity

The scavenging activity of the native or digested juice against 2,2′-azino-bis(3-ethylbenzothiazoline-6-sulfonic acid) radical action (ABTS^•+^) was estimated according to Re et al. [52]. Briefly, ABTS^•+^ was produced by reacting ABTS stock solution (7 mM) with 2.45 mM potassium persulfate and allowing the mixture to stand in the dark at at 22 °C for 14 h before use. Afterward, the ABTS^•+^ solution was diluted with ethanol to an absorbance of 0.7 ± 0.02 at 734 nm at 30 °C. Ten microlitres of native or digested juice was then mixed with 1.0 mL of diluted ABTS^•+^ solution, incubated at 30 °C for 6 min and the absorbance was measured at the same temperature using SPECTROstar Nano Microplate Reader (BMG LABTECH, Ortenberg, Germany) against distilled water. The control sample consisted of a solution prepared in the same manner but with distilled water instead of sample. The results were expressed as Trolox equivalent antioxidant capacity (TEAC, μM TE/mL) in the linear range of the standard 500–2000 μM/L (*R*^2^ = 0.9966).

#### 3.8.3. Ferric-Reducing Antioxidant Power

The FRAP assay was carried out according to the procedure of Benzie and Strain [53] with slight modification. The FRAP reagent was prepared fresh daily by mixing acetate buffer (300 mM, pH 3.6), 10 mM TPTZ (2,4,6-tri(2-pyridyl)-s-triazine) in 40 mM HCl, and 20 mM FeCl_3_ at 10:1:1 (*v/v/v*) ratio and was warmed to 37 °C prior to use. One hundred and fifty microliters of the native or digested juice were allowed to react with 2850 µL of the FRAP reagent at 37 °C for 4 min. The absorbance was then recorded at 593 nm against a blank solution prepared in the same manner but with distilled water instead of sample using SPECTROstar Nano Microplate Reader (BMG LABTECH, Ortenberg, Germany) and the results were expressed as Trolox equivalents (μM TE/mL) in the linear range of the standard 50–500 μM/L (*R*^2^ = 0.9970).

#### 3.8.4. Cupric Ion Reducing Antioxidant Capacity (CUPRAC) Assay

The CUPRAC assay was carried out according to the procedure of Apak et al. [54]. One mL of CuCl_2_ solution (1.0 × 10^−2^ M) was mixed with 1 mL of neo-cuproine methanolic solution (7.5 × 10^−3^ M), 1 mL of ammonium-acetate buffer solution (1 M, pH 7.0), and 0.1 mL of the native or digested juice followed by the addition of 1 mL distilled water (total volume = 4.1 mL). The mixture was then vortexed for 10 sec and incubated for 30 min at 22 °C. The absorbance was measured at 450 nm against a blank solution prepared in the same manner but with distilled water instead of sample using SPECTROstar Nano Microplate Reader (BMG LABTECH, Ortenberg, Germany). Trolox was used as a standard in the linear range 200–2000 μM/L (*R*^2^ = 0.9929) and the results were expressed as μM TE/mL.

### 3.9. Quantification of Phenolic Acids by HPLC-DAD

HPLC analysis of the phenolic acids was performed on an Elite LaChrome (Hitachi, Tokyo, Japan) chromatograph equipped with a pump L-2100 (Hitachi, Tokyo, Japan), a column oven L-2350 (Hitachi, Tokyo, Japan) and diode array detector (DAD) L-2455 (Hitachi, Tokyo, Japan). HPLC separation was performed by using a column Supelco Discovery HS C18 (5 μm, 250 × 4.6 mm, Sigma-Aldrich, St. Louis, MO, USA), operated at 30 °C under gradient conditions with mobile phase consisting of 2% (*v/v*) acetic acid (solvent A) and acetonitrile (solvent B) as reported by Mihaylova et al. [55]. The samples were filtered thought a 0.45 μm syringe filter (polytetrafluoroethylene filter) and 20 μL were injected into the system. The gradient program used was: 0–1 min, 95% A and 5% B; 1–40 min: 50% A and 50% B; 40–45 min: 100% B; 46–50 min: 95% A and 5% B. The detection of chlorogenic, caffeic, *p*-coumaric, and sinapic acids was carried out at 320 nm in the linear range 10–100 μg/mL for all the standards. The corresponding correlation coefficients were 0.9986, 0.9983, 0.9900, and 1.0000, respectively. The identification was done by comparing the retention time of the compound and those of the corresponding standard. The flow rate was 0.8 mL/min. The results were expressed in µg/100 g juice.

### 3.10. Total Carbohydrate Contents

The total sugar content of the juice samples and their digesta was estimated according to the phenol-sulfuric method [56]. Briefly, 0.1 mL of each sample was mixed with 1 mL of 5% phenol and 5 mL of sulfuric acid and placed in a water bath at 30 °C for 20 min. The absorbance was measured at 490 nm against blank with ultra-purified water instead of juice using SPECTROstar Nano Microplate Reader (BMG LABTECH, Ortenberg, Germany). The amount of presenting carbohydrates was determined from the calibration curve using glucose as a standard in a linear range of 20 to 100 μg/mL (*R*^2^ = 0.998) and the results were calculated as (g/100 g juice).

### 3.11. Quantification of Sugars and Polyols by HPLC-RID Method

The chromatographic separations and determination of sugars in the analyzed juices were performed on a high-performance liquid chromatograph HPLC Elite Chrome (Hitachi, Tokyo, Japan), equipped with a pump LC-20 AD, a column thermostat, refractive index detector (RID) Chromaster 5450 (Hitachi, Tokyo, Japan) and software. The separation was carried out on a Shodex^®^ Sugar SP0810 (7 μm, 300 × 8.0 mm i.d., Tokyo, Japan) column and a guard column Shodex SP -G (5 μm, 6 × 50 mm), operating at 85 °C, mobile phase ddH_2_O with flow rate 1.0 mL/min and injection volume of 20 μL as described by Petkova et al. [57]. The mobile phase was filtered under vacuum through a 0.2 μm membrane filter (Sartorius AG, Goettingen, Germany). All samples before injection were filtered through ISOLAB (Eschau, Germany) filters with a diameter of 4 mm and a pore size of 0.45 μm. Only juices containing chokeberry fruits were analyzed on the same column at a flow rate of 0.5 mL/min, column temperature 80 °C and RID operating temperature 35 °C [58]. The detection of sucrose, glucose, fructose and sorbitol was performed in the linear range of 0.5–10 mg/mL (sucrose-*R*^2^ = 0.9996, glucose-*R*^2^ = 0.9981, fructose-*R*^2^ = 0.9994, and sorbitol *R*^2^ = 0.9995). The identification was done by comparing the retention time of the analytes with those of the corresponding standard. The results were calculated using peak area and the values were presented for g/100 g juice.

### 3.12. Bioaccessibility Measurement

Bio-accessibility (%) was defined as the content of the compound released in the simulated digestion process compared to the content of the compound in the sample, and the value was calculated according to the Equation (3) [59]:

Bio-accessibility (%) = (C_f_/C_0_) × 100 (3)

where C_f_ is the final concentration of the compound (released in the simulated digestion) or activity and C_0_ is the initial concentration of the same compound or activity.

### 3.13. Statistical Analysis

All tests were carried out in triplicate and the results were presented as mean ± standard deviation (SD) using Microsoft Excel 2010.

Each experiment was performed in triplicate. The mean values for TPC, TFC, Anthocyanins and antioxidant activity (AOA), measured by four different methods (DPPH, FRAP, ABTS, CUPRAC), were calculated prior and after digestion. The correlation coefficient between TPC, TFC, Anthocyanins and AOA was calculated at each phase of digestion. To test for similarities among fruit juices, principal component analysis (PCA) was applied to the data set through multivariate exploratory techniques using XLSTAT software version 2018.1 (Addinsoft SARL, Paris, France). In the present study, 14 variables (TPC, TFC, Anthocyanins and AOA at two different stages of digestion) on a 14-dimensional space were finally mapped to seven principal components. Maximum variability was covered through first two components (in the range 88–92%) in all the analyses, which reflects that the outcome is produced without information loss.

## 4. Conclusions

The present study demonstrated that the studied fruit juices can be considered as a rich source of biologically active substances. In order to determine whether they remain active after passing through the digestive tract, the effect of in vitro digestion on the composition and content of sugars and phenolic substances was studied. The bioavailability of the tested compounds was affected by the digestive process and a significant decrease was reported. However, an exception to this trend was noted for some phenolic acids where liberation occurred in some digested samples. For all juices, total phenol content was found to contribute significantly to the AOA. Among them, pomegranate juice had the highest antioxidant activity, as well as the highest total content of phenols both before and after simulated digestion.

In conclusion, efforts should be put into juice stabilization prior to consumption in order to achieve better bio-accessibility of valuable compounds.

## Figures and Tables

**Table 1:** Physicochemical characteristics, individual and total sugar content of fruit juices prior to in vitro digestion **.

Sample/ Assay	pH	Dry Content, %	Sucrose, g/100 g	Glucose, g/100 g	Fructose, g/100 g	Sorbitol, g/100 g	Total Sugars, g/100 g
A	3.80	10.15	0.01 ± 0.01 ^e^	2.81 ± 0.04 ^c^	3.84 ± 0.01 ^b^	-	6.64 ± 0.06 ^d^
B	3.35	9.76	1.54 ± 0.01 ^b^	1.84 ± 0.01 ^f^	1.88 ± 0.02 ^e^	-	5.17 ± 0.02 ^e^
C	3.08	16.67	3.25 ± 0.04 ^a^	3.03 ± 0.05 ^b^	3.10 ± 0.03 ^c^	-	10.51 ± 0.09 ^a^
D	3.25	11.43	0.20 ± 0.02 ^d^	2.60 ± 0.04 ^d^	3.81 ± 0.03 ^b^	0.49 ± 0.01 ^c^	7.11 ± 0.03 *^,c^
E	3.45	10.89	0.02 ± 0.00 ^e^	2.89 ± 0.03 ^c^	1.78 ± 0.02 ^f^	0.58 ± 0.01 ^b^	4.69 ± 0.03 *^,f^
F	3.40	13.33	0.42 ± 0.02 ^c^	4.00 ± 0.02 ^a^	4.85 ± 0.01 ^a^		9.85 ± 0.02 ^b^
G	3.20	8.44	0.03 ± 0.00 ^e^	2.27 ± 0.02 ^e^	2.48 ± 0.01 ^d^	-	4.79 ± 0.02 ^f^
H	3.48	5.12	0.04 ± 0.01 ^e^	0.80 ± 0.01 ^g^	0.92 ± 0.01 ^g^	0.90 ± 0.02 ^a^	2.69 ± 0.01 *^,g^

* Data with sorbitol included. ** The data is presented as the mean (*n* = 3) ± S.D. Different letters within each column indicate significant differences between treatments according to Tukey’s test at *p* < 0.05.

**Table 2:** Phytochemical profile of fruit juices prior to in vitro digestion *.

Samples	Total Phenolic Contents (TPC),	Total Flavonoid Contents (TFC),	Total Monomeric Anthocyanins,
	mgGAE/mL	μgQE/mL	mg/L
A	15.56 ± 0.06 ^a^	910.4 ± 7.2 ^b^	26.67 ± 0.55 ^b^
B	3.45 ± 0.10 ^e^	815.62 ± 6.25 ^c^	0 ^f^
C	3.14 ± 0.03 ^e^	1667.71 ± 23.66 ^a^	0 ^f^
D	4.13 ± 0.06 ^d^	627.60 ± 3.61 ^e^	3.28 ± 0.05 ^d^
E	5.71 ± 0.08 ^c^	548.44 ± 34.80 ^f^	3.35 ± 0.11 ^d^
F	1.14 ± 0.02 ^f^	459.90 ± 11.84 ^g^	1.81 ± 0.03 ^e^
G	0.81 ± 0.01 ^f^	79.43 ± 3.39 ^h^	19.56 ± 0.53 ^c^
H	7.45 ± 0.33 ^b^	713.03 ± 9.15 ^d^	42.44 ± 0.98 ^a^

* The data is presented as the mean (*n* = 3) ± S.D. Different letters within each column indicate significant differences between treatments according to Tukey’s test at *p* < 0.05.

**Table 3:** Phenolic acids (µg/100 g juice) profile of fruit juices prior to in vitro digestion *.

Samples	Gallic Acid	Protocatechuic Acid	Chlorogenic Acid	Caffeic Acid	Ferulic Acid	*p*-Coumaric Acid	Sinapic Acid
A	0	0	1.25 ± 0.07 ^d^	1.54 ± 0.05 ^d^	˂LOQ	0 ^h^	2.31 ± 0.08 ^c^
B	0	˂LOQ	1.63 ± 0.03 ^c^	1.99 ± 0.02 ^c^	˂LOQ	3.92 ± 0.08 ^e^	2.22 ± 0.06 ^c^
C	0	0	6.78 ± 0.09 ^a^	0.75 ± 0.01 ^e^	0	12.23 ± 0.11 ^a^	8.45 ± 0.11 ^a^
D	˂LOQ	0	0 ^g^	3.25 ± 0.01 ^a^	˂LOQ	11.46 ± 0.11 ^b^	3.76 ± 0.04 ^b^
E	˂LOQ	˂LOQ	0 ^g^	1.61 ± 0.08 ^d^	˂LOQ	4.24 ± 0.05 ^d^	0.83 ± 0.01 ^e^
F	0	˂LOQ	0.73 ± 0.01 ^e^	1.27 ± 0.06 ^e^	˂LOQ	5.21 ± 0.11 ^c^	0.78 ± 0.01 ^f^
G	0	0	3.53 ± 0.109 ^b^	0.92 ± 0.02 ^g^	˂LOQ	3.15 ± 0.05 ^f^	0.70 ± 0.01 ^g^
H	0	˂LOQ	0 ^g^	2.70 ± 0.09 ^b^	˂LOQ	2.12 ± 0.02 ^g^	0.89 ± 0.02 ^d^

<LOQ–below limit of quantification. * The data is presented as the mean (*n* = 3) ± S.D. Different letters within each column indicate significant differences between treatments according to Tukey’s test at *p* < 0.05.

**Table 4:** Bio-accessibility (BA, %) of phytochemicals after in vitro digestion *.

Scheme	TPC		TFC		Total Monomeric Anthocyanins	
	mgGAE/mL	BA	μgQE/mL	BA	mg/L	BA
A	15.56 ± 0.06 ^a^	19.74	910.40 ± 7.2 ^b^	31.16	26.67 ± 0.55 ^b^	58.12
B	3.45 ± 0.10 ^e^	16.62	815.62 ± 6.25 ^c^	34.64	0 ^f^	0
C	3.14 ± 0.03 ^e^	16.29	1667.71 ± 23.66 ^a^	31.56	0 ^f^	0
D	4.13 ± 0.06 ^d^	26.20	627.60 ± 3.61 ^e^	24.25	3.28 ± 0.05 ^d^	0.93
E	5.71 ± 0.08 ^c^	13.52	548.44 ± 34.80 ^f^	26.98	3.35 ± 0.11 ^d^	0.89
F	1.14 ± 0.02 ^f^	26.49	459.90 ± 11.84 ^g^	67.00	1.81 ± 0.03 ^e^	1.11
G	0.81 ± 0.01 ^f^	13.83	79.43 ± 3.39 ^h^	35.53	19.56 ± 0.53 ^c^	50.36
H	7.45 ± 0.33 ^b^	17.34	713.03 ± 9.15 ^d^	24.54	0 ^f^	0

* The data is presented as the mean (*n* = 3) ± S.D. Different letters within each column indicate significant differences between treatments according to Tukey’s test at *p* < 0.05.

**Table 5:** Bio-accessibility (BA, %) of phenolic acids of fruit juices after in vitro digestion *.

Samples	Gallic Acid		Protocatechuic Acid		Chlorogenic Acid		Caffeic Acid		Ferulic Acid		*p*-Coumaric Acid		Sinapic Acid	
	µg/100 g	Bio-Accessibility (BA)	µg/100 g	BA	µg/100 g	BA	µg/100 g	BA	µg/100 g	BA	µg/100 g	BA	µg/100 g	BA
A	0 ^b^	-	0 ^b^	-	0.62 ± 0.03 ^e^	49.52	0.20 ± 0.01 ^c^	13.02	0	-	2.60 ± 0.08 ^c^	>100	0 ^f^	-
B	0 ^b^	-	0 ^b^	-	0.15 ± 0.01 ^f^	9.20	0 ^e^	-	0	-	1.56 ± 0.08 ^f^	39.80	0.67 ± 0.01 ^c^	30.18
C	0 ^b^	-	0 ^b^	-	1.36 ± 0.02 ^d^	20.06	0 ^e^	-	˂LOQ	NB	2.54 ± 0.02 ^d^	20.77	2.67 ± 0.02 ^a^	31.60
D	26.96 ± 0.23 ^a^	>100	0 ^b^	-	11.98 ± 0.55 ^b^	>100	0.23 ± 0.01 ^c^	6.99	0	-	4.07 ± 0.03 ^b^	35.54	1.42 ± 0.02 ^d^	37.69
E	0 ^b^	-	0 ^b^	-	8.80 ± 0.23 ^c^	>100	0.32 ± 0.02 ^b^	19.78	0	-	5.06 ± 0.04 ^a^	>100	2.00 ± 0.03 ^b^	>100
F	0 ^b^	-	20.70 ± 1.01 ^a^	>100	9.37 ± 0.21 ^c^	>100	0.99 ± 0.02 ^a^	77.95	0	-	2.64 ± 0.02 ^c^	50.67	1.11 ± 0.01 ^e^	>100
G	0 ^b^	-	0 ^b^	-	˂LOQ	NB	0 ^e^	-	0	-	0 ^g^	-	0 ^f^	-
H	0 ^b^	-	0 ^b^	-	67.44 ± 1.11 ^a^	>100	0.15 ± 0.01 ^d^	5.69	0	-	1.71 ± 0.02 ^e^	80.86	0 ^f^	-

* The data is presented as the mean (*n* = 3) ± S.D. Different letters within each column indicate significant differences between treatments according to Tukey’s test at *p* < 0.05.

## Data Availability

The data presented in this study are available on request from the corresponding author.

## Sample Availability

Not available.
